# Effect of photobiomodulation on the severity of oral mucositis and molecular changes in head and neck cancer patients undergoing radiotherapy: a study protocol for a cost-effectiveness randomized clinical trial

**DOI:** 10.1186/s13063-019-3196-8

**Published:** 2019-02-01

**Authors:** Allisson Filipe Lopes Martins, Túlio Eduardo Nogueira, Marília Oliveira Morais, Angélica Ferreira Oton-Leite, Marize Campos Valadares, Aline Carvalho Batista, Nilceana Maya Aires Freitas, Cláudio Rodrigues Leles, Elismauro Francisco Mendonça

**Affiliations:** 1 Department of Oral Pathology, Dentistry Faculty, Federal University of Goiás, Avenida Universitária Esquina com 1ª Avenida, s/n. Setor Universitário, Goiânia, Goiás CEP 74605-220 Brazil; 2 Department of Prevention and Oral Rehabilitation, Dentistry Faculty, Federal University of Goiás, Avenida Universitária Esquina com 1ª Avenida, s/n. Setor Universitário, Goiânia, Goiás CEP 74605-220 Brazil; 3 Department of Stomatology, Araujo Jorge Cancer Hospital , R. 239, 206—Setor Universitário, Goiânia, Goiás CEP 74175-120 Brazil; 4 Department of Pharmacology and Cellular Toxicology, Pharmacy Faculty, Federal University of Goiás, 5ª Avenida Esquina com Rua 240, s/n. Setor Universitário, Goiânia, Goiás CEP 74605-170 Brazil; 5 Department of Radiotherapy, Araujo Jorge Cancer Hospital, R. 239, 206–Setor Universitário, Goiânia, Goiás CEP 74175-120 Brazil

**Keywords:** Cost-effectiveness, Oral mucositis, Head and neck cancer, Inflammatory mediators, Oxidative stress

## Abstract

**Background:**

Oral mucositis (OM) is the most frequent and debilitating acute side effect associated with head and neck cancer (HNC) treatment. When present, severe OM negatively impacts the quality of life of patients undergoing HNC treatment. Photobiomodulation is a well-consolidated and effective therapy for the treatment and prevention of severe OM, and is associated with a cost reduction of the cancer treatment. Although an increase in the quality of life and a reduction in the severity of OM are well described, there is no study on cost-effectiveness for this approach considering the quality of life as a primary outcome. In addition, little is known about the photobiomodulation effects on salivary inflammatory mediators. Thus, this study aimed to assess the cost-effectiveness of the photobiomodulation therapy for the prevention and control of severe OM and its influence on the salivary inflammatory mediators.

**Methods/design:**

This randomized, double-blind clinical trial will include 50 HNC patients undergoing radiotherapy or chemoradiotherapy. The participants will be randomized into two groups: intervention group (photobiomodulation) and control group (preventive oral care protocol). OM (clinical assessment), saliva (assessment of collected samples) and quality of life (Oral Health Impact Profile-14 and Patient-Reported Oral Mucositis Symptoms questionnaires) will be assessed at the 1st, 7th, 14th, 21st and 30th radiotherapy sessions. Oxidative stress and inflammatory cytokine levels will be measured in the saliva samples of all participants. The costs are identified, measured and evaluated considering the radiotherapy time interval. The incremental cost-effectiveness ratio will be estimated. The study will be conducted according to the Brazilian public health system perspective.

**Discussion:**

Photobiomodulation is an effective therapy that reduces the cost associated with OM treatment. However, little is known about its cost-effectiveness, mainly when quality of life is the effectiveness measure. Additionally, this therapy is not supported by the Brazilian public health system. Therefore, this study widens the knowledge about the safety of and strengthens evidence for the use of photobiomodulation therapy, providing information for public policy-makers and also for dental care professionals. This study is strongly encouraged due to its clinical relevance and the possibility of incorporating new technology into public health systems.

**Trial registration:**

Brazilian Registry of Clinical Trials—ReBEC, RBR-5h4y4n. Registered on 13 June 2017.

**Electronic supplementary material:**

The online version of this article (10.1186/s13063-019-3196-8) contains supplementary material, which is available to authorized users.

## Background

The association of chemotherapy (CT) and radiotherapy (RT) is the most frequent treatment approach for head and neck cancer (HNC) in cases of locally advanced disease [1]. Oral mucositis (OM) is the main side effect of RT and chemoradiation therapy (CRT) for HNC, its severity dependent on the number of RT sessions [2–6]. OM is an inflammatory reaction that can affect the entire oral cavity and gastrointestinal tract [7–10]. Intense pain, the need for nutritional supplementation, a higher number of clinical appointments and treatment interruption are common events associated with severe OM. These side effects may influence the survival rate and increase the costs of the HNC treatment [3, 5, 11–14].

CT and RT increase the levels of reactive oxygen species that may cause upregulation of transcription factors, such as NF-κB and STAT3 [4]. This increased level of transcription factors activates the production of inflammatory cytokines, such as matrix metalloproteinase, leading to tissue damage [4, 5, 15]. The use of photobiomodulation therapy (PBMT) has been encouraged and is described as effective therapy for OM treatment [16, 17]. Recent data showed that PBMT improves quality of life (QoL) and is effective in the control of OM, reducing the morbidity and costs associated with OM [16–24]. Oberoi et al. [18] concluded that PBMT reduced the number of severe OM episodes and pain associated with it. However, several protocols for PBMT have been described with differences in the laser wavelength, time of irradiation, frequency and energy used. They suggested that research should be conducted investigating the ideal parameters and clinical viability.

Although PBMT has been used for the treatment of CRT-induced OM, the mechanism of action of this therapy in the stressed oral cavity is not completely understood. Animal studies showed that the reduction of OM severity might be due to a reduction of COX-2 levels and the neutrophilic infiltrate in the wound; besides that, PBMT may also promote collagen organization, resulting in ulcer healing [25, 26]. Silva et al. [21] studied the effects of PBMT on the inflammatory mediators and oxidative stress of CT-induced OM. They showed that PBMT can enhance interleukin-10 levels, an anti-inflammatory cytokine that reduces the damage caused by neutrophils and macrophages, and suggested that interleukin-6 has an important role in OM. Salvador et al. [27] showed that PBMT can reduce the levels of interleukin-8 but did not find any differences in the antioxidant proteins in patients undergoing hematopoietic stem cell transplantation. Although these studies are about CT-induced OM, the initiation process of OM appears to be the same in CT-induced and RT-induced OM [4]; thus, the influence of PBMT on the levels of inflammatory cytokines may be similar.

Only a few studies have investigated the effects of PBMT on the inflammatory and oxidative stress proteins in CRT-induced OM. Oton-Leite et al. [28] showed that use of a red laser is associated with a reduction in inflammation and repair, evoking a less intense inflammatory response. In this specific study, patients were irradiated three times a week, and the authors suggested that additional studies with different protocols should be conducted to better understand the effects of PBMT in OM.

According to a study conducted in the USA in 2002, OM was associated with an incremental cost of US$1700–6000 in the HNC treatment, due to an increased resource use, hospital days, opioid use and other factors [13]. Nonzee et al. [11] reported an incremental cost of US$18,515 due to severe degrees of OM in lung cancer patients. The use of PBMT is associated with a reduction in the costs of severe OM. Bezinelli et al. [19] reported a reduction of 30% of the costs in patients undergoing hematopoietic stem cell transplantation who received PBMT to prevent OM.

Antunes et al. [29] conducted a cost-effectiveness study regarding the use of PBMT in the treatment of OM, taking into consideration the public health system perspective and the prevention of severe OM as the effectiveness measure. The authors concluded that the patients treated with PBMT presented an incremental cost of US$1689.00, when compared with the patients not exposed to PBMT; the incremental cost-effectiveness ratio was US$4961.37 per severe case of OM prevented. To our knowledge, there has not been a study that has evaluated the cost-effectiveness of PBMT for the control and treatment of OM based on patient-reported outcomes. This type of study may allow a comparison between the different technologies and drugs used for the treatment of OM.

Thus, the aim of this study is to assess the cost-effectiveness and molecular effects of PBMT in the treatment and prevention of severe OM. The hypothesis is that PBMT is more costly but more effective than conventional treatment of OM and that PBMT modulates the inflammatory and oxidative stress proteins. This study will contribute to a better understanding of the PBMT mechanism and may allow the laser technology to be consolidated and used in public health systems, providing a better QoL for patients undergoing HNC treatment.

## Methods/design

**Fig. 1:**
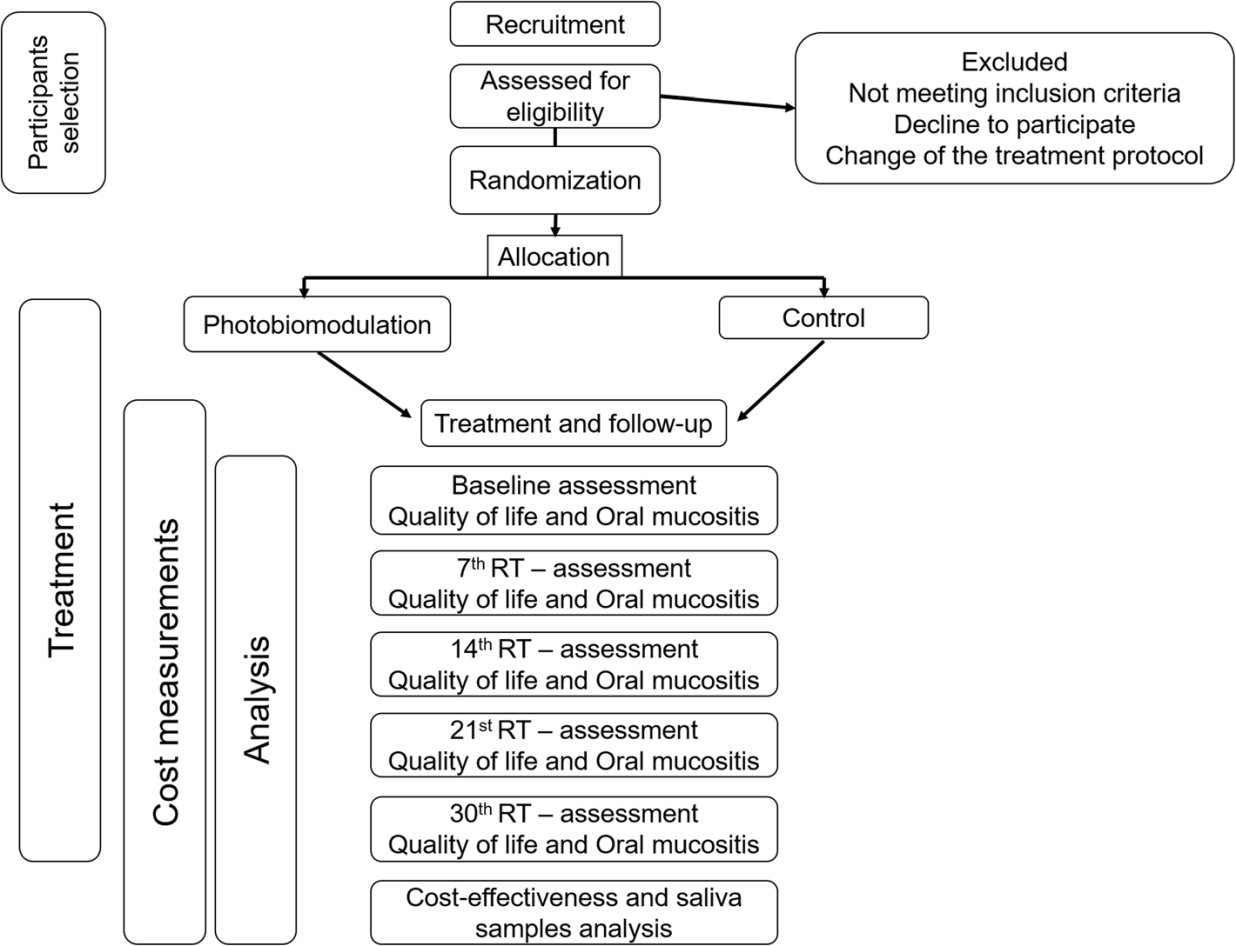
Flowchart of the study. RT radiotherapy

**Fig. 2:**
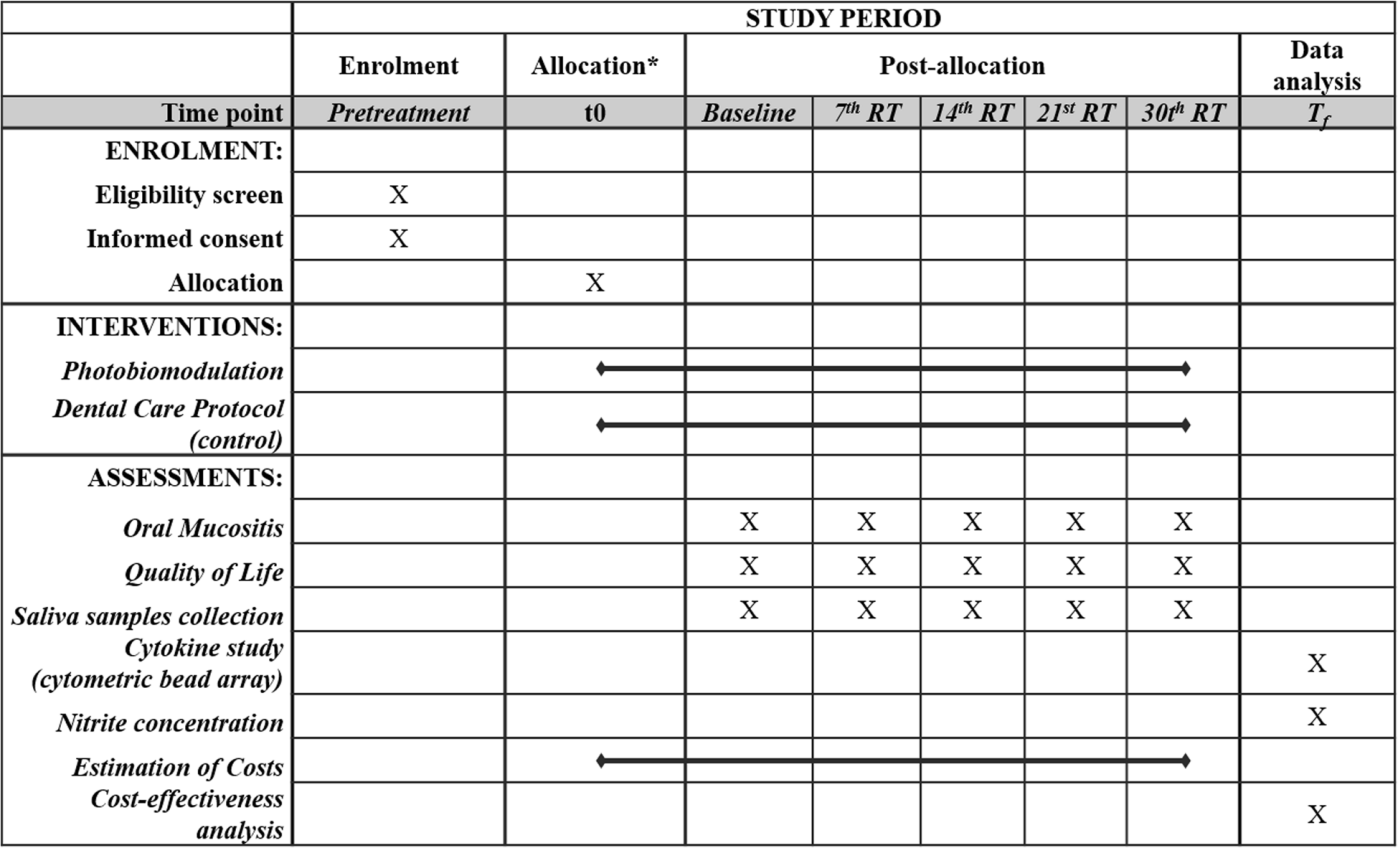
Timeline of the study. *The allocation period and the baseline evaluation will be performed at the same appointment. RT radiotherapy

This study is a randomized, double-blind clinical trial that will be conducted in Araujo Jorge Cancer Hospital, Goiânia, Goiás, Brazil and the Faculty of Dentistry of the Federal University of Goiás, Goiânia, Goiás, Brazil. The study was approved by the Research Ethics Committee of the Federal University of Goiás and the Research Ethics Committee of the Araujo Jorge Cancer Hospital (Numbers 2.608.604/2018 and 2.131.323/2017, respectively). Moreover, the study was registered in the Brazilian Registry of Clinical Trials (RBR-5h4y4n). All participants who accepted to participate in the study signed a written informed consent form. Figure 1 shows the flowchart of the study and Fig. 2 presents the outcomes and time points according to recommendations for interventional trials (SPIRIT). A SPIRIT checklist is presented in Additional file 1.

### Participants

The participants will be HNC patients of the Brazilian public health system—Brazilian Unified Health System (Sistema Único de Saúde (SUS))—who will be treated with RT, with or without CT, in Araujo Jorge Hospital, Goiânia, Goiás, Brazil. To estimate the study sample size, we used the OpenEpi online calculator (http://www.openepi.com). The 95% significance level (1 – α error type I), a study power of 80% (1 – β error type II) and a 1.0 ratio of unexposed/exposed were set as estimator parameters. Finally, we considered a prevalence of severe OM of 60% for the control group and 21% for the PBMT group (OR = 0.18) [18]. The calculated minimum sample size was 50 participants (25 per group) and an additional 10% was considered to compensate for possible loss to follow-up.

Individuals older than 18 years of age of both sexes will be included in the study, edentulous or not, with neoplastic lesions in the head and neck site (oral cavity, oropharynx, nasopharynx and advanced stage laryngeal tumors; T3 N1 or T3 N2 and T4 with any N status). All participants will be subjected to RT (with or without CT), with a minimum dose of 50 Gy, 2 Gy/day and five times per week, in the conventional scheme of RT. All patients will be treated with the Elekta Compact (Elekta AB, Stockholm, Sweden), a linear accelerator that generates photon radiation with fixed energy of 6 MV. Participants will be excluded if they present with an infection or salivary gland tumors, Sjögren’s syndrome, are submitted to palliative treatment or were previously treated with RT and/or CT in the head and neck area. Individuals diagnosed with lymphoma, melanoma or skin cancer will also be excluded.

In this hospital setting, every HNC patient who will be submitted to RT must be evaluated by the Dental Care Service to obtain authorization before starting the HNC treatment. On this occasion, the individuals will be invited to participate in the study. After acceptance, the principle researcher will obtain a signed written informed consent form from the participants. Participants will be excluded from the trial in cases of death, withdrawal or absence from five or more laser therapy sessions.

### Randomization and blinding/masking

Participants will be randomly assigned into two groups: group 1 (PBMT, five times per week) and group 2 (control). A computer-based random number generator (www.randomizer.org) will be used to assign the participants to the study groups. Simple randomization will be performed with an allocation ratio of 1:1. The sequence will be generated by an independent collaborator. The letters A and B will be used to determine the group: A = intervention and B = control. The sequence will be concealed in consecutively numbered opaque envelopes. Each envelope will be opened at the time of the first RT session. The participants will be blinded to the allocation group. In the control group, a sham laser will be used; the participants will hear the characteristic sound of the equipment but no irradiation will be performed. The researcher responsible for assessing the OM will also be blind regarding the study group and will not have contact with the participants since the assessment will be carried out using the photographs of the participants.

### Study groups

#### Group 1: photobiomodulation therapy

The participants in this group will be submitted to laser therapy five times a week, during the RT. The PBMT protocol will begin in the first RT session, be conducted before each RT session and continue until the end of the treatment. Since the RT sessions will be scheduled in a specific hour, it will be possible to control participant appointments. If severe OM develops at the end of the RT, the patient will receive additional sessions.

The Twin Flex Evolution Laser (MMOptics, São Paulo, Brazil) will be used at a wavelength of 660 nm (red laser), 25 mW of power, in continuous mode, with punctual and perpendicular application in contact with the mucosa. The transferred energy will be 6.2 J/cm^2^ for 10 s on a spot area of 0.04 cm^2^, and thus energy of approximately 0.25 J will be deposited at each point. Before each session, the tip of the laser will be disinfected with a solution of 70% alcohol and wrapped in a plastic film. The laser beam will not be irradiated in a malignant lesion smaller than 1 cm if observed, or near a surgical site.

Specific anatomical regions be considered for the PBMT: right and left buccal mucosa (10 points on each side), upper and lower labial mucosa (four points on each lip), hard palate (three points), lateral surface of the tongue (10 points on each side), soft palate (three points), dorsal tongue (three points), floor of the mouth (two points) and labial commissure (one point on each side). This protocol is based on a previous study by Oton-Leite et al. [30].

The participants will also be submitted to a dental care protocol that consists of elimination of the infection focus, a fluoride rinse, a chlorhexidine 0.12% rinse three times a day diluted in water in a 1:1 proportion, recommendation to drink at least 1.5 L of water per day and the maintenance of good oral hygiene. If the presence of OM is observed, an oral ointment with hydrocortisone 5 mg, neomycin 5 mg, troxerutin 20 mg, ascorbic acid 0.5 mg and benzocaine 2 mg will be prescribed (Gingilone; Farmasa, São Paulo, Brazil).

#### Group 2: control group

The participants in the control group will have laser therapy appointments; however, they will not be irradiated. A simulation of the irradiation will be conducted by turning on the equipment, which produces the characteristic sound of the laser device, but the laser will not be irradiated. As mentioned for the PBMT group, if participants present with severe OM at the end of treatment, they will be submitted to PBMT. The participants will also be submitted to laser therapy in cases of RT suspension due to severe OM, during the suspension interval, to speed up the healing process.

The participants in the control group will be submitted to the dental care protocol. This protocol consists of elimination of the infection focus, a fluoride rinse, a chlorhexidine 0.12% rinse three times a day diluted in water in a 1:1 proportion, a recommendation to drink at least 1.5 L of water and the maintenance of good oral hygiene. In the presence of OM, Gingilone (Farmasa), an oral ointment with hydrocortisone 5 mg, neomycin 5 mg, troxerutin 20 mg, ascorbic acid 0.5 mg and benzocaine 2 mg, will be used. At the end of the RT treatment, the participants in this group will also receive PBMT if needed.

### Outcomes

The effectiveness of the treatment will be measured based on severe OM being prevented and by the impact on the QoL of the participants. The levels of inflammatory cytokines in saliva will also be investigated.

#### Oral mucositis evaluation

An assessor who is blinded to the treatment will conduct the OM evaluation. The researcher responsible for the PBMT will take intraoral pictures and save them on a hard disk for later evaluation. The evaluation will be in the first appointment and after the 7th, 14th, 21st and 30th RT sessions. The photographs will be taken using a T3i camera, with a circular flash (MR-14EX II) and a lens of Ef-s 18–55 mm F/3.5–5.6 (Canon, Tokyo, Japan). Pictures of the upper and lower labial mucosa, buccal mucosa, hard and soft palate, lateral surface of the tongue and floor of mouth will be acquired.

For the OM classification, the World Health Organization (WHO) [31] and the National Cancer Institute (NCI) [32] classifications will be used.

According to the WHO scale, the OM is classified as: 0, no signs or symptoms; 1, oral soreness and erythema; 2, erythema, ulcer, both solid and liquid diets tolerated; 3, ulcers and liquid diet only; 4, oral alimentation is impossible. The NCI scale is a graduated scale as follows: 0, no visible alterations; 1, presence of erythema; 2, noncontiguous ulcers up to 1.5 cm in diameter; 3, contiguous ulcers larger than 1.5 cm in diameter; 4, ulcers showing necrosis and bleeding. In this study, OM will be considered severe when classified with grades 3 or 4 according to the NCI and/or WHO scales.

#### Quality of life

The translated, adapted and validated Brazilian version [33] of the Oral Health Impact Profile, in its simplified form (OHIP-14) [34], will be used to assess the oral health-related quality of the participants. This questionnaire contains 14 questions, divided into seven subscales: functional limitation, physical pain, physiological discomfort, physical disability, physiological disability, social disability and handicap. The questions will be answered on a 5-point Likert scale, the responses will be summarized and the final score will be calculated.

The translated version of the Patient-Reported Oral Mucositis Symptoms (PROMS) scale will also be used to assess the OM effects reported by the patient [35]. This scale was originally used to assess OM due to CT; however, its use in patients undergoing RT is also feasible [36, 37]. The scale contains 10 questions, which are answered on a 10-cm uninterrupted visual analog scale. The participant will be asked to indicate the score for each item. The sum of the 10 items will be used as the PROMS score.

These instruments will be applied during the first appointment and after the 7th, 14th, 21st and 30th RT sessions based on previous studies [23, 38] that also evaluated the QoL weekly. As reported earlier, the OM evaluation will occur at the same time points. The questionnaires will be applied before the PBMT session and if the participant is not able to answer, a calibrated healthcare professional will provide help.

#### Saliva collection and analysis

The collection of saliva will be performed before initiating the HNC treatment and after the 7th, 14th, 21st and 30th RT sessions, based on the method of Oton-Leite et al. [28] and Navazesh [39]. The participants will be instructed not to eat or drink 1 h before the collection of saliva.

The collection of unstimulated saliva will be performed in an artificial light environment, with the participants sitting with their eyes open, with their head slightly tilted down, without talking, opening their mouth or swallowing the saliva during the time of collection. Initially, the participants will be instructed to wash their mouth with water and swallow any saliva in their mouth. The participants will then be instructed not to swallow for a period of 5 min in order to accumulate saliva. This accumulated saliva will be deposited in a sterile Falcon-type millimeter tube.

The sample will be centrifuged for 15 min at 4500 rpm. The samples will then be transferred to another tube and diluted (1:1) in PBS solution (0.4 mM NaCl and 10 mM NaPO_4_) containing protease inhibitors (0.1 mM phenylmethylsulfonyl fluoride, 0.1 mM benzethonium chloride, 10 mM EDTA and 0.01 mg/ml aprotinin A) and 0.05% Tween-20. The solution will be homogenized, distributed in aliquots of approximately 2 ml and frozen at − 80 °C. The concentrations of inflammatory cytokines, nitrite and total proteins will be assessed in each saliva sample.

#### Inflammatory study

##### Cytokine study

The concentrations of the cytokines (IL-1β, IL-10, IL-6, IL-8, IL-12, TNF-α) in the saliva samples will be determined by cytometric bead array (CBA). The CBA method enables the detection of multiple soluble mediators from a relatively small sample, using bead-based flow cytometric immunoassays, since beads of different sizes or colors are used. Thus, this assay measures the levels of the six cytokines in a small sample of saliva [40].

The cytokines will be measured by a BD FACSCanto II flow cytometer (BD Biosciences, San Jose, CA, USA) using the CBA human inflammatory cytokines kit (BD Biosciences) following the manufacturer’s recommendations. Briefly, the Human Chemokine Standards will be reconstituted and serially diluted in the Assay Diluent. After this, the Human Chemokine Capture Beads will be mixed and a 10-μl aliquot of each Capture Bead, for each assay tube to be analyzed, will be placed into a single tube. A 96-well plate will be used to perform the assay. The initial wells will be filled with 50 μl of the Human Chemokine Standard; then, 50 μl of the saliva sample will be placed, followed by the addition of 50 μl of the Human Chemokine PE detection reagent. The saliva and detection reagents will be incubated for 3 h, protected from light. After this, 100 μl of wash buffer will be added to each well and centrifuged at 1100 rpm for 5 min. The supernatant will be carefully aspirated and discarded. Finally, the bead pellet will be resuspended with the addition of 150 μl of wash buffer. The values will be determined based on the negative control and a standard curve.

The Bradford method will be used to measure the total protein concentration in the saliva samples, expressed as milligrams per milliliter. This concentration will be used to adjust the salivary cytokine values for each sample. The values of the cytokine levels in saliva samples corrected for total proteins will be expressed as picograms per milligrams of protein.

##### Nitrite concentration

The concentration of nitrite in the saliva samples will be measured using the method described by Green et al. [41]. A volume of the saliva sample will be transferred to a 96-well plate and then the same volume of the Griess reagent will be added to each well. After 10 min, the absorbance will be measured by a spectrophotometry reader (SpectraMax 340; Molecular Devices) using a 570-nm filter. A standard curve will be used to calculate the nitrite concentrations.

### Economic analysis

#### Study perspective and setting

The cost-effectiveness evaluation will be performed from the health provider perspective and conducted alongside the randomized clinical trial. The setting considered was the Dental Care Service of the hospital, which includes a dental office and a waiting room, and the staff comprise a dentist licensed in PBMT and a dental assistant.

#### Estimation of costs

##### PBMT costs

Cost estimation will include the direct costs of resources associated with the treatment of OM during RT. All fixed and variable costs will be identified, measured and valuated for each cost item. The variable costs will include consumable items (70% ethanol, gauzes, procedure gloves, etc.). The fixed costs will include the income of the professionals and costs associated with the laser equipment. For the equivalent annual cost (EAC) of durable goods, a discount rate of 5% and a usage life of 5 years will be considered for calculation, using the following formula:

$$ \mathrm{EAC}=\left(n\times x\right)/\left(1-1/\left[{\left(1+n\right)}^t\right]\right) $$

where EAC = annual cost equivalent, *t* = useful life, *x* = purchase price and *n* = discount index.

The average cost per PBMT session will be calculated as the sum of the variable and fixed costs. The cost of staff will consider the value of the work hours proportional to the time spent in each session: (Monthly wage / monthly working time) × time spent.

The data on costs of consumables will be obtained from the administrative department of the hospital and the cost of the laser equipment will be based on mean market prices. Costs will be estimated in Brazilian currency (Brazilian Real (BRL)) and later converted to international dollars using the purchasing power parity exchange rate.

The costs of RT and CT and those related to the routine preventive oral health protocol will not be considered since they are similar for both groups. Besides that, the social costs related to wider societal costs (e.g., loss of productivity resulting from treatment, family costs, etc.) will not be considered. Similarly, other capital costs or those associated with implementation of the dental care service will not be included.

##### Costs due to severe OM

The costs related to OM will include episodes of severe OM (WHO degrees III and IV), hospitalizations, use of opioid analgesics and parenteral nutrition. These data will be retrieved from the hospital records and cost estimation will be based on the governmental database for the public health system.

### Cost-effectiveness analysis

The incremental cost-effectiveness ratio (ICER) will be calculated considering the incidence of severe degrees of OM as the measure of effectiveness for the PBMT and control groups. We will also measure the effectiveness based on patient-reported outcomes (including QoL measures). A decision tree-type diagram will be created to represent all clinically relevant events.

The ICER will be calculated to determine the cost-effectiveness of PBMT. The ICER will be calculated by dividing the cost difference (Cost2 – Cost1) by the difference between the frequency of severe OM in both groups. The ICER will represent the incremental cost to avoid one case of severe OM using PBMT.

Additionally, another ICER will be calculated by dividing the cost difference (Cost2 – Cost1) by the difference between the QoL in both groups. The ICER represents the incremental cost to avoid the loss of 1 point on the QoL measure, assessed using the difference between the last evaluation (30th RT) and the baseline (0 RT). Two different ICERs will be calculated considering the scores of each instrument (OHIP-14 and PROMS). The following formula summarizes the ICER analysis.

$$ \mathrm{ICER}=\left(\mathrm{Cost}2-\mathrm{Cost}1\right)/\left(\mathrm{QoL}2-\mathrm{QoL}1\right) $$

Univariate sensitivity analysis will be performed to assess the uncertainty in the parameters associated with PBMT.

### Statistical analysis of data

The comparison of the clinical and demographic characteristics of the participants between the two groups will be performed using the Pearson chi-square test for categorical variables. The Mann–Whitney test will be used to compare the severity of OM and concentration of cytokines between the two groups. The Friedman test will be used to compare changes in outcomes in the different time points for each of the treatment groups. In case of nonrejection of the null hypothesis, a post-hoc power analysis will be performed to test whether the study has sufficient power to identify the differences between groups. Values of *p* < 0.05 will be considered statistically significant. The statistical analysis will be performed using the IBM SPSS 20.0 statistical package (SPSS Inc., Chicago, IL, USA).

## Discussion

This is the first cost-effectiveness study to include patient-reported outcomes to assess the effectiveness of PMBT in the treatment and prevention of severe OM in patients undergoing HNC treatment. The inclusion of patient-reported outcomes, such as oral health-related QoL, is important in cost-effectiveness studies because this allows comparisons between different technologies. Although PBMT is one of the most effective therapies for OM, other treatments have been studied; for example, the use of keratinocyte growth factor, date palm pollen, gamma-d-glutamyl-l-tryptophan, pilocarpine hydrochloride, superoxide dismutase mimetic and ATL-104 mouthwash [42–51]. In a recent literature review, Cinausero et al. [51] discussed several strategies to treat and manage OM, ranging from basic oral hygiene to physical strategies, including PBMT and natural medicines. Since several treatments are available for OM management, patient-reported outcomes, like the ones that will be evaluated in this study, allow for comparisons between the cost-effectiveness of different treatments suggested for OM. Besides that, the investigation of inflammatory cytokines and oxidative stress in the saliva of the participants will contribute to the elucidation of the mechanism of action in OM associated with chemoradiation.

Shariati et al. [52] pointed out that the lack of attention to the field of dentistry from public policy-makers discourages economic studies, and effective and beneficial health services are neglected because of this. This study aims to consolidate the use of PBMT to treat and prevent severe OM, mainly in the public health system, which currently does not provide any treatment for this condition but only for the pain associated with it. Adequate resource allocation and cost reduction are expected from the results of this study, as well as an improvement in the QoL of the HNC patients.

Cost-effectiveness studies about OM treatment are rare [29, 53]. Antunes et al. [29] found that the ICER was US$4961.37 per severe OM case prevented, also considering the SUS. However, the authors did not investigate any patient-reported outcomes, such as QoL, as we propose in this study. Elad and Thierer [53] studied the cost-effectiveness of topical chlorhexidine in decreasing the risk of mortality, and OM was an important outcome evaluated. The authors found that chlorhexidine mouthwash was more effective and less costly than not using it. However, the expected outcomes were analyzed throughout a static model using secondary data that had been previously published, carried out considering hematologic patients.

Currently, several PBMT protocols are considered when treating OM [18]. The energy per spot and the laser schedule are the most important parameters that differ among the studies. The red laser is most frequently used and presents favorable results. In our study, a diode laser will be used with a wavelength of 660 nm (red laser), 25 mW of power and 0.24 J of energy per point. The PBMT will be performed daily until the end of the HNC treatment. This protocol was used by Oton-Leite et al. [30] and satisfactory results were achieved.

In a recent study, Sonis et al. [54] discussed the effect of PBMT on tumor growth, proliferation, local invasion, metastases and the treatment response of the tumor. Based on the results of the studies that suggested upregulation and downregulation of genes and pathways associated with these aspects, and the lack of strong evidence regarding the safety of use of PBMT, the authors discouraged the use of PBMT as a treatment for OM. However, to date, the Multinational Association of Supportive Care in Cancer and the International Society of Oral Oncology still consider and recommend PBMT as an intervention for OM [55]. Use of PBMT is also associated with a lower frequency of interruption of the oncological therapy, and this may play an important role in the prognosis and chance of cure for the patient [24]. Corroborating these studies, Antunes et al. [56] showed that HNC patients who were submitted to PBMT presented a tendency of better overall and disease-free survival when compared with patients submitted to placebo. Additionally, the progression-free survival was significantly higher among patients submitted to PBMT and a better response to HNC treatment was observed. We believe it is important to study the effects of PBMT on inflammatory mediators and oxidative stress, so that a safety protocol and guideline can be established and proposed. Additionally, the diversity of PBMT protocols reported in the literature make it difficult to discuss the results from the different studies.

Finally, the effectiveness of PBMT is well established in the literature. This investigation aims to establish a safety evidence-based protocol for the use of PBMT in the treatment and prevention of severe OM, mainly in the public health system. The results of the investigation will be important to public policy-makers since they may enhance understanding regarding the costs involved in OM treatment and prevention with PBMT, favoring better resource allocation. Additionally, the investigation is also important to HNC patients since the results will encourage the incorporation of PBMT into the public health system, resulting in a better QoL for the patients during treatment.

## Trial status

The trial started recruitment and treatment of participants in August 2017.

## Additional file

Additional file 1: SPIRIT 2013 checklist. (PDF 159 kb)

## Abbreviations

CRT: Chemoradiation therapy
CT: Chemotherapy
EAC: Equivalent annual cost
HNC: Head and neck cancer
ICER: Incremental cost-effectiveness ratio
*n*: Discount index
NCI: National Cancer Institute
OHIP-14: Oral Health Impact Profile-14
OM: Oral mucositis
PBMT: Photobiomodulation therapy
PROMS: Patient-Reported Oral Mucositis Symptoms
QoL: Quality of life
RT: Radiotherapy
SUS: Brazilian Public Health System
*t*: Useful life
WHO: World Health Organization
*x*: Purchase price
